# A Rare Case of Gastric Aberrant Pancreas Causing Bleeding from a Gastric Ulcer

**DOI:** 10.70352/scrj.cr.25-0520

**Published:** 2026-02-07

**Authors:** Tsuyoshi Saito, Hirotaka Miyai, Ryutaro Kato, Ryosuke Niwamoto, Shuhei Ueno, Masahiro Kimura, Shuji Takiguchi

**Affiliations:** 1 Department of Gastroenterological Surgery, Kariya Toyota General Hospital, Kariya, Aichi, Japan; 2 Department of Gastroenterological Surgery, Nagoya City University Graduate School of Medical Sciences, Nagoya, Aichi, Japan

**Keywords:** aberrant, pancreas, submucosa

## Abstract

**INTRODUCTION:**

An aberrant pancreas is pancreatic tissue lacking anatomical and vascular continuity with the normal pancreas. It typically occurs in the gastrointestinal tract, especially in the stomach, duodenum, and small intestine. Most cases are asymptomatic and discovered incidentally during imaging or surgery. Gastric aberrant pancreas rarely presents with bleeding, making diagnosis and treatment decisions challenging.

**CASE PRESENTATION:**

A 34-year-old man presented with epigastric pain and melena. Endoscopy at a local hospital revealed a submucosal tumor (SMT) with active bleeding at the greater curvature of the stomach. After admission, further imaging and endoscopic ultrasonography showed a 20-mm low-echo mass in the submucosa and muscularis propria with ulceration. Histopathology of a biopsy confirmed aberrant pancreatic tissue. Due to progressive anemia from ulcer bleeding, laparoscopic partial gastrectomy was performed. The SMT was completely resected, and the postoperative course was uneventful. Pathological examination confirmed a Heinrich type I aberrant pancreas.

**CONCLUSIONS:**

Most cases of aberrant pancreas are asymptomatic; however, in this case, a preoperative diagnosis of gastric submucosal aberrant pancreas was made by endoscopic examination and puncture aspiration cytology because of the relatively rare bleeding symptoms. In this case, laparoscopic partial gastrectomy was performed to control bleeding.

## Abbreviations

EUS-FNA
: endoscopic ultrasound-guided fine-needle aspiration
SMT
: submucosal tumor

## INTRODUCTION

An aberrant pancreas is the pancreatic tissue that lacks anatomical continuity with the pancreas and has blood circulation that is separate from that of the normal pancreas. Its mechanism of development is thought to be by the ectopic migration of part of the dorsal pancreatic rudiment of the duodenum during the fetal period in areas such as the gastrointestinal tract, biliary duct, liver, spleen, or mediastinum. However, the majority of such tissues are located in the stomach, duodenum, or small intestine.^1)^ Patients with an aberrant pancreas are usually asymptomatic; therefore, such an entity is typically discovered incidentally during endoscopy, surgery, or autopsy.

Gastric aberrant pancreas is the 2nd most common type of aberrant pancreas after the duodenum; however, it is rarely detected due to bleeding. Here, we describe a case of laparoscopic local gastrectomy after endoscopic hemostasis of a gastric aberrant pancreas that caused bleeding from a gastric ulcer.

## CASE PRESENTATION

**Fig. 1:**
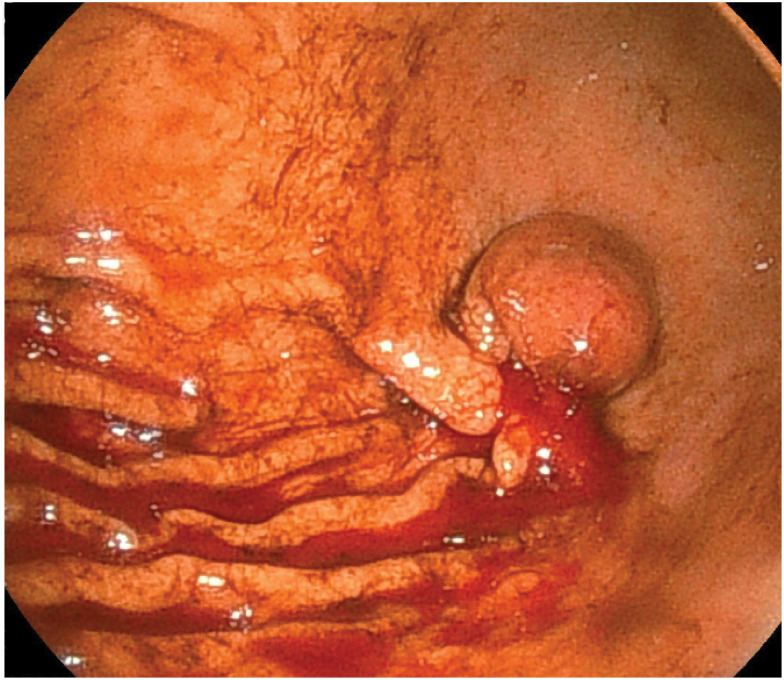
Esophagogastroduodenoscopy findings. A submucosal tumor is found in the greater curvature of the gastric angle, and bleeding is observed from an ulcer at the rise of the oral hilum (esophagogastroduodenoscopy performed at a local hospital).

A 34-year-old man with no remarkable medical history presented with epigastralgia and melena. At a local hospital, esophagogastroduodenoscopy revealed an SMT with an active bleeding ulcer at the greater curvature of the stomach (**Fig. 1**). The patient was then referred to our hospital and admitted.

**Fig. 2:**
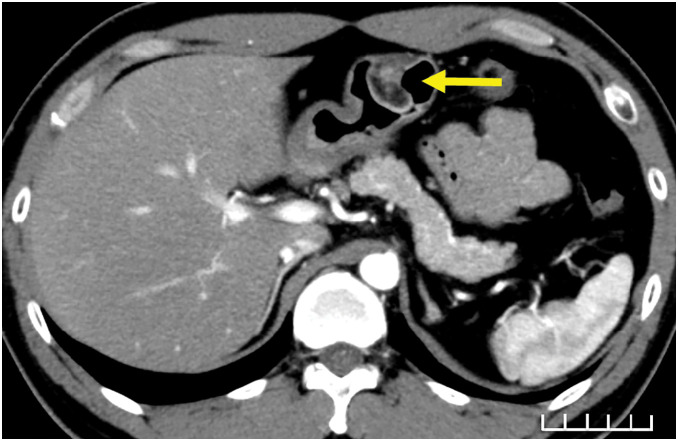
Axial CT scan of the abdomen. A 20-mm-sized nodule is observed in the greater curvature of the gastric horn, which is contrast-enhanced from the early stage, and a low-absorption area is observed inside the nodule. A mucosal defect is observed, which is suspected to be an ulcer (arrow).

**Fig. 3:**
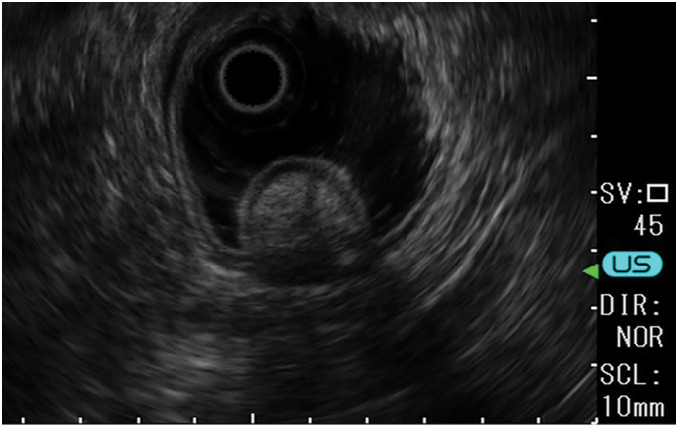
Endoscopic ultrasonography image. The lesion appears as a well-defined hypoechoic mass with the 3rd and 4th layers as the main parts. The lesion is a well-demarcated, hypoechoic mass with 3rd and 4th layers, internal dots of hyperechoic and duct-like echoes, and thickening of the 4th layer.

Laboratory examinations revealed mild anemia but no other significant findings. On esophagogastroduodenoscopy, active bleeding from the peptic ulcer stopped. CT showed a 20-mm nodule with early contrast in the greater curvature of the stomach (**Fig. 2**). During the follow-up period, endoscopic ultrasonography revealed that the tumor was a low-echo mass located in the submucosa and muscularis propria (3rd and 4th layers). Thickening of the muscularis propria surrounding the mass was also observed. The tumor measured 20 mm, and a peptic ulcer was located at the base of the SMT (**Fig. 3**). Histopathological examination after biopsy of the peptic ulcer revealed gastric aberrant pancreas.

**Fig. 4:**
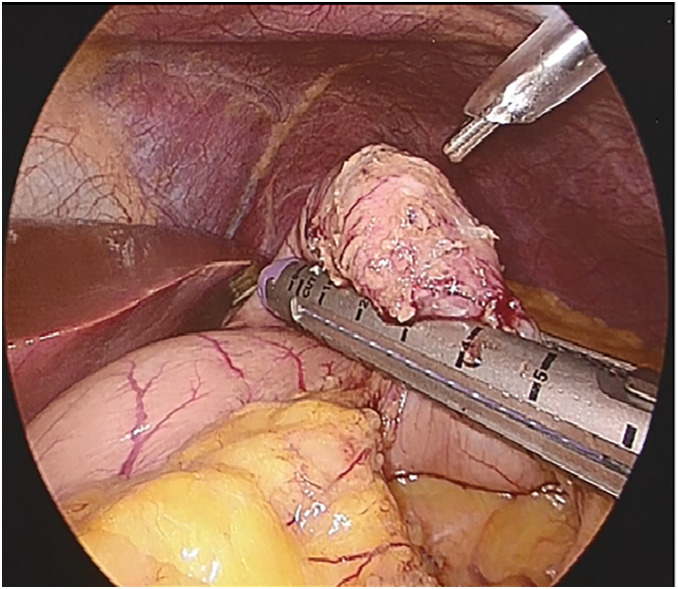
Operative findings The tumor was carefully resected with adequate surgical margins by sandwiching the lesion between normal tissues, using 2 firings of a 60-mm linear stapler.

**Fig. 5:**
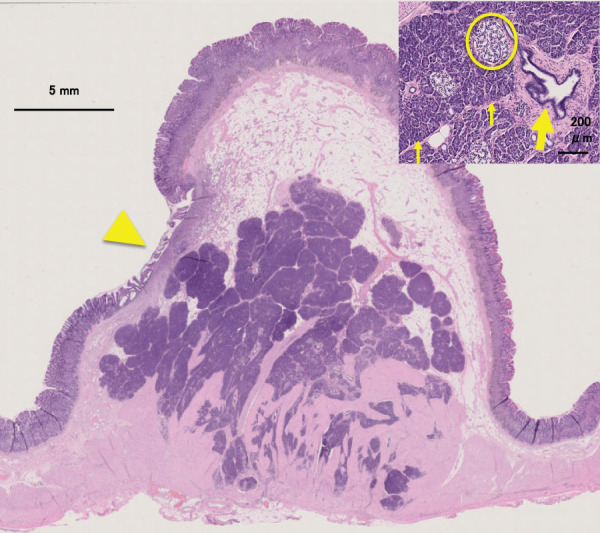
Histopathological findings. The pancreatic lobules and ducts differentiated from the submucosa to the muscularis propria are enlarged, and ulceration is observed in some areas (arrowhead). Fibrosis is observed immediately below the ulcer; however, hemorrhage or disrupted blood vessels are not observed. No malignancies are observed. Abundant adipose tissue is distributed in the submucosal layer above the tumor. Growth of acinar cells (thin arrow), excretory ducts (thick arrow), and islets of Langerhans (circle). Stain: hematoxylin and eosin

Laparoscopic partial gastrectomy was performed as it was necessary for gastric SMT resection because of progressive anemia due to bleeding from the peptic ulcer. Observation of the abdominal cavity revealed an SMT in the greater curvature of the stomach, without exposure to the serosal surface. To identify the full extent of the tumor, the greater omentum was divided along the gastric wall to open the omental bursa. The tumor contour was easily recognized by gentle grasping with forceps; therefore, intraoperative endoscopy was not utilized. The tumor was resected using a linear stapler. The specimen was retrieved in a specimen bag, opened intraoperatively, and macroscopically confirmed to have been completely resected before concluding the procedure (**Fig. 4**). The postoperative course was uneventful, and the patient was discharged on the 5th POD. The SMT measured 25 × 18 × 20 mm and showed severe ulcerative changes. The pathological diagnosis was aberrant pancreas with islets of Langerhans, acinar cells, and excretory ducts (**Fig. 5**).

## DISCUSSION

Aberrant pancreas is found at a site where the pancreatic tissue is anatomically different from the pancreas, and was first reported by Schultz et al. in 1727 (as cited in Elfving and Hästbacka).^2)^ According to a report by De Castro Barbosa et al., of the 471 cases of aberrant pancreas, 27.7% were in the duodenum, 25.5% in the stomach, and 15% in the jejunum.^3)^ The submucosa, muscularis mucosa, and serosa of the stomach account for 73%, 17%, and 10% of cases, respectively, with the submucosa accounting for the largest proportion.^4)^

In pathology, Heinrich’s classification is used for aberrant pancreas. Type I is composed of acinar cells, conduits, and islets of Langerhans; Type II is composed of acinar cells and conduits; and Type III is composed of conduits only.^5)^ In the present case, the aberrant pancreas consisted of acinar cells, conduits, and islets of Langerhans, and was diagnosed as Heinrich type I.

CT and ultrasonography are often used to diagnose aberrant pancreas; however, Zhang et al. reported that only 14% of 184 cases of aberrant pancreas could be diagnosed using ultrasonography before surgery or biopsy.^6)^ However, some reports state that the diagnosis of aberrant pancreas using ultrasound endoscopy with puncture aspiration cytology is useful, with a positive diagnosis rate ranging from 50% to 80%.^7)^ In our case, we performed ultrasonographic endoscopic puncture aspiration cytology and collected the pancreatic adenohypophysis from the gastric submucosa before surgery, allowing us to make a histological diagnosis.

Among aberrant pancreatic lesions, those with symptoms are extremely rare (approximately 6%), and even if symptoms are present, there is no specific relationship with the localization site.^8)^ It has been reported that 20.9% of aberrant pancreatic lesions are symptomatic when limited to the upper gastrointestinal tract.^9)^ Symptomatic aberrant pancreas localized in the upper gastrointestinal tract has been reported to cause abdominal pain in more than half of the cases, as well as gastric discomfort, abdominal distention, vomiting, and bleeding. It has been reported that the percentage of patients with aberrant pancreas and bleeding symptoms is only 8%–9%.^9)^ To identify surgically treated cases of bleeding gastric aberrant pancreas, we performed a comprehensive literature search using the Ichushi-Web database with the Japanese keywords “gastric aberrant pancreas,” “gastric ectopic pancreas,” and “bleeding,” as well as PubMed using the keywords “gastric heterotopic pancreas,” “gastric aberrant pancreas,” “gastric ectopic pancreas,” “bleeding,” and “hemorrhage.” Based on this search, including the present case, a total of 16 cases in patients aged ≥20 years were identified in the literature as far as could be ascertained. The patients ranged in age from 22 to 67 years, with a marked male predominance. Most lesions were located in the gastric antrum or gastric horn (**Table 1**).^9–23)^ Preoperative definitive diagnosis by EUS-FNA was achieved in only a few recent cases, whereas the majority were diagnosed postoperatively. Surgical procedures varied from partial gastrectomy to distal subtotal gastrectomy, with minimally invasive approaches increasingly adopted in recent years. Histopathologically, Heinrich type I was the most common subtype, followed by types II and III. The present case represents one of the few cases in which a definitive preoperative diagnosis was established by EUS-FNA and successful treatment was achieved with laparoscopic partial gastrectomy. In the present case, the lesion consisted of normal Heinrich type I pancreatic tissue, and ulceration was observed in the overlying gastric mucosa. Several mechanisms have been proposed to explain gastric mucosal injury associated with aberrant pancreas. Alkaline secretion from the pancreatic tissue may stimulate gastrin release, resulting in increased gastric acid and pepsin secretion and subsequent development of mucosal lesions, such as ulcers.^24)^ Alternatively, chronic inflammation arising from aberrant pancreatic tissue may induce edema and congestion in the gastric submucosa, with congestion of fragile submucosal vessels potentially leading to bleeding and diapedesis into the gastric lumen.^25)^ In this case, the bleeding is presumed to have occurred in association with these mechanisms.

**Table 1:** Reported cases of bleeding gastric aberrant pancreas treated surgically

No	Author	Year	Age (years)	Sex	Location	Definitive diagnosis by EUS-FNA	Surgical method	Pathological type
1	Hudock^10)^	1956	38	M	Antrum	No	Distal subtotal gastrectomy	Unknown
2	Takebayashi^11)^	1986	35	M	Antrum	No	Distal gastrectomy	Heinrich type I
3	Shaked^12)^	1989	30	M	Antrum	No	Distal gastrectomy	Unknown
4	Yamasaki^13)^	2003	47	M	Lower body	No	Laparoscopic partial gastrectomy	Heinrich type I
5	Sanada^14)^	2007	48	M	Gastric angle	No	Laparoscopic partial gastrectomy	Heinrich type I
6	Teke^15)^	2007	54	F	Antrum	No	Distal subtotal gastrectomy	Heinrich type I
7	Tagata^16)^	2014	22	M	Antrum	No	Partial gastrectomy	Heinrich type I
8	Kuroda^17)^	2015	50	M	Antrum	No	LECS	Heinrich type III
9	Yagi^18)^	2017	66	M	Antrum	No	Laparoscopic distal gastrectomy	Heinrich type II
10	Chamberlain^19)^	2019	47	M	Gastric body	No	Laparoscopic gastric wedge resection	Heinrich type I
11	Nakajima^20)^	2019	67	M	Upper body	Yes	LECS	Unknown
12	Matsubara^21)^	2020	43	M	Gastric angle	Yes	Open partial gastrectomy	Heinrich type I
13	Obana^22)^	2021	59	M	Antrum	Yes	CLEAN-NET	Heinrich type II
14	LeCompte^9)^	2022	37	M	Unknown	No	Laparoscopic gastric wedge resection	Unknown
15	Raghavendra^23)^	2025	62	M	Gastric body	No	Robotic local excision	Heinrich type II
16	Present case	2025	34	M	Gastric angle	Yes	Laparoscopic partial gastrectomy	Heinrich type I

CLEAN-NET, combination of laparoscopic and endoscopic approaches to neoplasia with non-exposure technique; EUS-FNA, endoscopic ultrasound-guided fine-needle aspiration; F, female; LECS, laparoscopic and endoscopic cooperative surgery; M, male

Since an aberrant pancreas is usually seen incidentally and is often asymptomatic, the indications for surgery should be carefully considered. However, in 85% of symptomatic aberrant pancreas cases, surgical or endoscopic resection has been reported to eliminate or improve symptoms.^9)^ Therefore, we conclude that surgical treatment is necessary to prevent bleeding. However, even if the patient is asymptomatic, resection may be indicated if gastric cancer, gastric submucosal tumor, or malignant aberrant pancreas cannot be ruled out as differential diseases.^26)^

## CONCLUSIONS

Most cases of aberrant pancreas are asymptomatic; however, in the present case, the patient presented with the relatively rare symptom of bleeding, and a preoperative diagnosis of gastric submucosal aberrant pancreas was established using endoscopic examination and fine-needle aspiration cytology. Laparoscopic partial gastrectomy was performed to control the bleeding. Given that gastric aberrant pancreas generally has a lower malignant potential compared with gastrointestinal stromal tumor, preoperative diagnosis is clinically valuable as it allows for appropriate surgical planning. Specifically, it helps to minimize the risk of tumor dissemination associated with gastric content spillage during tumor manipulation and avoids the selection of excessively invasive procedures that would be justified only for malignant lesions. Therefore, accurate preoperative identification of aberrant pancreas can guide the choice of an appropriately tailored, minimally invasive surgical approach while maintaining patient safety.

## DECLARATIONS

### Availability of data and materials

Data sharing is not applicable to this article as no datasets were generated or analyzed during the current study.

## References

[1] Watanabe T, Aoyagi K, Tomioka Y, et al. Endoscopic ultrasonography of duodenal aberrant pancreas: comparison with histology after endoscopic resection. J Med Ultrason (2001) 2015; 42: 277–80. 26576585 10.1007/s10396-014-0592-2

[2] Elfving G, Hästbacka J. Pancreatic heterotopia and its clinical importance. Acta Chir Scand 1965; 130: 593–602. 5865465

[3] De Castro Barbosa JJ, Dockerty MB, Waugh JM. Pancreatic heterotopia; review of the literature and report of 41 authenticated surgical cases, of which 25 were clinically significant. Surg Gynecol Obstet 1946; 82: 527–42. 21024692

[4] DeBord JR, Majarakis JD, Nyhus LM. An unusual case of heterotopic pancreas of the stomach. Am J Surg 1981; 141: 269–73. 7457747 10.1016/0002-9610(81)90172-0

[5] Heinrich HV. Ein Beitrag zur Histologie des sogen. akzessorischen Pankreas (in German). Virchows Arch 1909; 198: 392–401.

[6] Zhang Y, Sun X, Gold JS, et al. Heterotopic pancreas: a clinicopathological study of 184 cases from a single high-volume medical center in China. Hum Pathol 2016; 55: 135–42. 27195908 10.1016/j.humpath.2016.05.004

[7] Wiersema MJ, Vilmann P, Giovannini M, et al. Endosonography-guided fine-needle aspiration biopsy: diagnostic accuracy and complication assessment. Gastroenterology 1997; 112: 1087–95. 9097990 10.1016/s0016-5085(97)70164-1

[8] Dolan RV, ReMine WH, Dockerty MB. The fate of heterotopic pancreatic tissue. A study of 212 cases. Arch Surg 1974; 109: 762–5. 4420439 10.1001/archsurg.1974.01360060032010

[9] LeCompte MT, Mason B, Robbins KJ, et al. Clinical classification of symptomatic heterotopic pancreas of the stomach and duodenum: a case series and systematic literature review. World J Gastroenterol 2022; 28: 1455–78. 35582670 10.3748/wjg.v28.i14.1455 PMC9048474

[10] Hudock JJ, Wanner H, Reilly CJ. Acute massive gastro-intestinal hemorrhage associated with pancreatic heterotopic tissue of the stomach. Ann Surg 1956; 143: 121–5. 13275909 10.1097/00000658-195601000-00017 PMC1464949

[11] Takebayashi J, Hashimoto N. Acute gastric hemorrhage associated with pancreatitis occurring in gastric aberrant pancreas tissue: report of a case (in Japanese with English abstract). Gastroenterol Endosc 1986; 28: 807–11.

[12] Shaked G, Maor E. Ectopic pancreas in the gastric wall with massive bleeding (in Hebrew with English abstract). Harefuah 1989; 116: 358–9. 2737561

[13] Yamasaki T, Nebiki H, Aomatsu K, et al. A case of gastric aberrant pancreas causing gastrointestinal bleeding (in Japanese). Nihon Shokakibyo Gakkai Zasshi 2003; 100: 667–72. 12833860

[14] Sanada K, Shibata M, Sugihara K. A case of gastric aberrant pancreas causing hemorrhagic shock (in Japanese with English abstract). Nihon Rinsho Geka Gakkai Zasshi 2007; 68: 54–7.

[15] Teke Z, Kabay B, Kelten C, et al. Ectopic pancreas of the gastric antrum contiguous to a gastrointestinal stromal tumor manifesting as upper gastrointestinal bleeding: report of a case. Surg Today 2007; 37: 74–7. 17186352 10.1007/s00595-006-3340-4

[16] Tagata T, Hamano T, Teramoto H, et al. A case of gastric aberrant pancreas with bleeding and diagnosed by endoscopic ultrasonography. Endosc Ultrasound 2014; 3(Suppl 1): S7. PMC4569934 26425534

[17] Kuroda K, Fujii M, Shirasaka D, et al. Successful resection of a gastric aberrant pancreas causing gastrointestinal bleeding by laparoscopy and endoscopy cooperative surgery (in Japanese with English abstract). Nihon Shokakibyo Gakkai Zasshi 2015; 112: 515–21. 25759226 10.11405/nisshoshi.112.515

[18] Yagi N, Tomizawa N, Arakawa K. An ectopic pancreas causing pyloric stenosis followed by rupture and intraperitoneal bleeding (in Japanese with English abstract). J Abdom Emerg Med 2017; 37: 1081–5.

[19] Chamberlain P, Prabhu A, Bednar F. Gastric hemorrhage caused by heterotopic pancreas. J Gastrointest Surg 2019; 23: 1940–1. 30761466 10.1007/s11605-019-04144-w

[20] Nakajima T, Akatsuka M, Kitamura Y, et al. Iishosei sui ga utagaware fukukukyo-naishikyo godo shujutsu ni te setsujo shita shukketsusei i kaiyo no 1 rei (in Japanese). Meiwa Igaku Journal 2019; 6: 38–41.

[21] Matsubara K, Ishida M, Morito T, et al. A rare case of enlarged gastric heterotopic pancreas with retention cysts: a case report and literature review. Int J Surg Case Rep 2020; 74: 284–8. 32773294 10.1016/j.ijscr.2020.07.035 PMC7503788

[22] Obana Y, Kanehira E, Inoue H, et al. A case of gastric aberrant pancreas with ulcer formation and bleeding treated by CLEAN-NET (in Japanese with English abstract). J Jpn Soc Endosc Surg 2021; 26: 235–41.

[23] Raghavendra D, Jebakumar SGS, Muthiah J, et al. Diagnostic dilemma in gastroduodenal lesions: heterotopic pancreas mimicking gastrointestinal stromal tumours managed by minimally invasive approach – a case series and review of the literature. J Minim Access Surg 2025; doi:10.4103/jmas.jmas_42_25. PMC12904626 41351162

[24] Benner WH. Diagnostic morphology of aberrant pancreas of the stomach; report of five cases. Surgery 1951; 29: 170–81. 14817618

[25] MADINAVEITIA JM, MADINAVEITIA M, LOMA V. Páncreas aberrante [Aberrant pancreas] (in Spanish). Rev Esp Enferm Apar Dig Nutr 1951; 10: 31–42. 14844971

[26] Christodoulidis G, Zacharoulis D, Barbanis S, et al. Heterotopic pancreas in the stomach: a case report and literature review. World J Gastroenterol 2007; 13: 6098–100. 18023108 10.3748/wjg.v13.45.6098 PMC4250899

